# Interactions of Tenofovir, Lamivudine, Abacavir and Didanosine in Primary Human Cells

**DOI:** 10.3390/pharmaceutics3020326

**Published:** 2011-06-22

**Authors:** Omar Janneh, Saye H. Khoo

**Affiliations:** 1 Department of Biomolecular and Sport Sciences, Coventry University, Priory Street, Coventry CV1 5FB, UK; 2 Department of Pharmacology and Therapeutics, The University of Liverpool, 70, Pembroke Place, Block H, First Floor, Liverpool. L69 3GF, UK

**Keywords:** tenofovir, lamivudine, abacavir, didanosine, drug transport

## Abstract

Certain triple nucleoside/tide reverse transcriptase inhibitor (NRTI) regimens containing tenofovir (TDF) have been associated with rapid early treatment failure. The mechanism is unknown, but may be at the level of drug transport. We measured the lipophilicity of the drugs [^3^H]-lamivudine (3TC), -didanosine (ddI), -TDF and -ABC. Peripheral blood mononuclear cells (PBMCs) were used to evaluate drug–drug interactions at the level of drug transport. PBMCs were measured for the expression of P-glycoprotein (P-gp), multidrug resistance-associated protein-1 (MRP-1) and breast cancer resistance protein (BCRP) by flow cytometry. The rank order of the lipophilicity of the drugs were ABC⋙3TC≥ddI>TDF. The accumulation of [^3^H]-3TC, -ddI and -TDF were temperature sensitive (suggesting facilitated transport), in contrast to [^3^H]-ABC. ABC reduced the accumulation of [^3^H]-3TC, and cell fractionation experiments suggested this was mainly in membrane-bound [^3^H]-3TC. ABC/TDF and ABC/ddI increased the accumulation of [^3^H]-3TC and 3TC/TDF also increased the accumulation of [^3^H]-TDF. In contrast, none of the NRTI/NtRTI incubations (alone or in combination) altered the accumulation of [^3^H]-ABC and -ddI. PBMC expression of P-gp, MRP1 and BCRP were detected, but none correlated with the accumulation of the drugs. The high failure rates seen with TDF, ABC and 3TC are not fully explained by an interaction at transporter level.

## 1. Introduction

Tenofovir disoproxil fumarate (TDF) is a potent nucleotide reverse transcriptase inhibitor (NtRTI) widely used in combination antiretroviral therapy. However, several studies have reported excessive and rapid early treatment failure in triple nucleoside reverse transcriptase inhibitor (NRTI) regimens containing TDF, lamivudine (3TC), didanosine (ddI), abacavir (ABC), (3TC+ddI or ABC+3TC) [1,2] or in non-nucleoside reverse transcriptase inhibitor (NNRTI)-containing regimens with TDF+ddI as backbone in patients with high baseline viraemia [3-5].

Phenotypic susceptibility to the drug combinations is reduced with the development of the K65R mutation in HIV-1 reverse transcriptase. Although the K65R mutation is rarely selected as compared with the thymidine analogue mutations associated with stavudine and zidovudine, the use of TDF+ABC, TDF+ddI and ABC+d4T in combination with 3TC or emtricitabine [6] is to be avoided due to the high levels of treatment failures.

Although the explanation for these high failure rates is unclear, overlapping resistance profiles, variable drug permeation into target cells, esterase cleavage of TDF, drug–drug and drug-food interactions at the level of influx/efflux transports may contribute to the acquisition of resistance. NRTIs and NtRTIs are prodrugs requiring intracellular phosphorylation to their active metabolites. Consequently, cellular entry of the drugs is important for successful inhibition of viral replication. Intracellular accumulation of a drug is dependent on several factors, including lipophilicity, ion trapping, protein binding, and drug transporters. Generally, the transport of most nucleoside analogues is mediated by one or more of the following nucleoside transporter systems: concentrative nucleoside transporters, (Na^+^-dependent), equilibrative nucleoside transporters (Na^+^-independent) and H^+^/peptide transporters [7]. Nevertheless, P-glycoprotein (P-gp), multi-drug resistance proteins (MRPs), breast cancer resistance protein (BCRP) and influx transporters such as human organic cation and anion transporters (hOCTs/hOATs) may mediate the transport of nucleoside and nucleotide analogues. There is some evidence that some NRTI/NtRTIs are substrates of P-gp, BCRP and MRPs [8-19], but there is no evidence that the efflux of ddI and TDF are BCRP-mediated. In other studies, the activities of P-gp and MRP were inhibited by ABC, 3TC and TDF [20-22]. However, evidence suggests that ddI does not inhibit P-gp activity and that 3TC is also not a substrate of P-gp [23]. There is evidence for the involvement of hOATs and hOCTs in the intracellular accumulation of some NRTI/NtRTIs [14,24-26]. However, while TDF, ddI and 3TC are devoid of any BCRP inhibitory effect, ABC inhibits BCRP [21].

We postulate that suboptimal concentrations as a consequence of drug–drug interactions at the level of transport may favour the emergence of drug resistant viruses. Here, the effects of the drug lipophilicity and drug transporter expression on the transport and cell-associated concentrations of [^3^H]-3TC, -ddI, -TDF and -ABC were evaluated in primary human cells (which express transporters [27,28]) in the absence or presence of unlabelled interacting NRTI/NtRTI (singly or in combination).

## 2. Materials and Methods

### 2.1. Reagents

Blood buffy coats were purchased from the North West and North Wales Regional Blood Services (Liverpool, UK). [^3^H]-3TC, -ddI, -TDF and -ABC (specific activities 8.0 Ci/mmol, 41.0 Ci/mmol 3.4 Ci/mmol and 2 Ci/mmol, respectively) were purchased from Moravek Biochemicals, Inc, CA, USA. CEM, CEM_VBL_ and CEM_E1000_ cell lines were from Dr R Davey (Royal North Shore Hospital, St Leonards, NSW 2065, Australia) and U937 cells obtained from Porton Down (Salisbury, UK). Mouse anti-human IgG_2A_ (IgG_2A_ and IgG_2A_:rPE) and IgG_1_ isotype control antibodies were purchased from Serotec Ltd (Oxford, UK). Mouse anti-human P-gp antibody (UIC2:rPE) was obtained from Immunotech (Marseilles, France). Mouse anti-human BCRP (BXP-21) and MRP1 specific mouse anti-human primary antibody QCRL-1 were obtained from Abcam Ltd (Cambridge, UK) and Monosan antibodies (NL) respectively. Secondary goat anti-mouse IgG conjugated to rPE and FITC were obtained from Serotec (Oxford, UK) and Sigma respectively. All other reagents unless otherwise stated were purchased from Sigma Chemical Co (Poole, UK).

### 2.2. Octanol-saline partition coefficient

Since the cellular association of a drug within target cells is a composite of the physicochemical properties of the drug (passive diffusion, ion trapping), and active influx/efflux, we measured the lipophilicity of each drug ([^3^H]-3TC, [^3^H]-ddI, [^3^H]-TDF, [^3^H]-ABC and [^14^C]-mannitol (as the positive control) as described previously [24] to establish the contribution of lipophilicity to drug uptake. Briefly, an equal volume of octanol and phosphate buffered saline (PBS) were presaturated by vigorously vortexing them for 5 min. The suspension was allowed to settle for 10 min before the upper saturated octanol layer was carefully removed and stored in a separate tube. Then [^3^H]-3TC (10 nM), -ddI (15 nM), -TDF (15 nM), -ABC (10 nM) and [^14^C]-mannitol (31 nM) were then diluted with 3 mL of PBS. Then 540 μL aliquots of each compound were added to an equal volume of presaturated octanol and vortexed for 5 min. The samples were centrifuged (1000 g for 5 min). The concentrations of radiolabelled drug in a volume of the octanol (upper layer) and in a similar volume of saline (lower layer) were measured using scintillation counting. The octanol-saline partition coefficient was determined as a ratio of radiolabelled drug in the octanol phase to radiolabelled drug concentration in the saline phase.

### 2.3. Isolation of Peripheral Blood mononuclear cells (PBMCs)

11 PBMC samples were isolated from blood buffy coats using Lymphoprep (Alexis-sheild, Oslo, Norway), following the manufacturer's instructions. An aliquot of the PBMC samples were cryopreserved in fetal calf serum containing 10% dimethyl sulphoxide for batch analysis of membrane proteins by flow cytometry.

### 2.4. Drug–drug interaction studies of [^3^H]-ABC, -3TC, -ddI and -TDF and effects of the unlabelled drugs

A series of preliminary transport experiments were performed to look at the concentration-ranging (0-100 μM) effects of the unlabelled drugs on cell-associated concentrations of the labelled drugs in T-lymphoblastoid cell lines (CEM(parental), CEM_VBL_ (P-gp-overexpressing) and CEM_E1000_ (MRP1-overexpressing) and the monocytic cell line U937. The CEM and its variant cells have been also shown to express hOATs [28].

We also used 50 μM dipyridamole, 100 μM of deoxycytidine (dC), deoxyinosine (dI) and deoxyguanosine (dG) as positive controls to inhibit the transport of the drugs. The concentrations used of these inhibitors were based on previous observations [27,29,30]. The isolated PBMCs (5 × 10^6^ cells) were incubated with 1 μM of [^3^H]-3TC, -ddI, -TDF or -ABC in RPMI 1640 medium containing 10% fetal calf serum at 4 °C or 37 °C, for 30–45 min in the absence or presence of fixed concentrations (50 μM) of the interacting unlabelled drugs (3TC, ddI, TDF, and ABC). In a limited number of PBMC samples, we investigated the effects of two drug combinations on the uptake of labelled compounds as described above. The incubations were terminated by centrifugation (15,000 g, 1 min at 0 °C). A-100 μL aliquot of the supernatant was counted for radioactivity and the cell pellets were washed three times in ice-cold phosphate buffered saline followed by rapid centrifugation before the pellets were solubilised in 100 μL of distilled water as described previously [31] and counted for radioactivity. Data were expressed as cellular association ratio (CAR), this being the ratio of the amount of radiolabelled drugs associated with the cell pellets to the amount in a similar volume of supernatant after incubation; cell volume of each PBMC being 0.4 pL [32].

In a separate experiment, cells were incubated with the tritiated compounds ([^3^H]-3TC, -ddI and -TDF) in the absence or presence of 50 μM ABC as described. The assay was terminated by rapid centrifugation in a chilled microcentrifuge and the cell pellets were washed three times in ice-cold PBS as described, followed by three times of rapid freezing in liquid nitrogen and thawing at room temperature. The suspension was layered on top of 1 mL of ice-cold 42% percoll solution containing 0.25 M sucrose, 1.5 mM magnesium chloride, pH 7.1, and centrifuged (12,000 g, 30 min at 4 °C). After centrifugation, the radioactivity in the clear supernatant (assumed as intracellular drug) and in the pellet/debris, harvested from the bottom of the gradient (assumed as membrane-bound drug) were determined by scintillation counting.

### 2.5. Flow cytometric analysis of membrane proteins for P-gp, MRP1 and BCRP

PBMC expression of P-gp, MRP1 and BCRP were performed on the frozen samples as previously described [33,34].

#### Ethics

No ethical approval was required in the collection and use of the blood products from the blood transfusion services.

### 2.6. Statistical analysis

Data were expressed as mean ± S.D. Shapiro-Wilk test was used to assess the distribution of the data, followed by Kruskal-Wallis test to allow multiple comparisons of drug-treated samples to respective controls. In each case, significance between control and drug-treated means was assumed if *P* < 0.05. Analyses were performed using Statsdirect statistical software version 2.3.1, 2003 (StatsDirect LTD, Cheshire, UK).

## 3. Results and Discussion

### 3.1. Results

**Figure 1.**
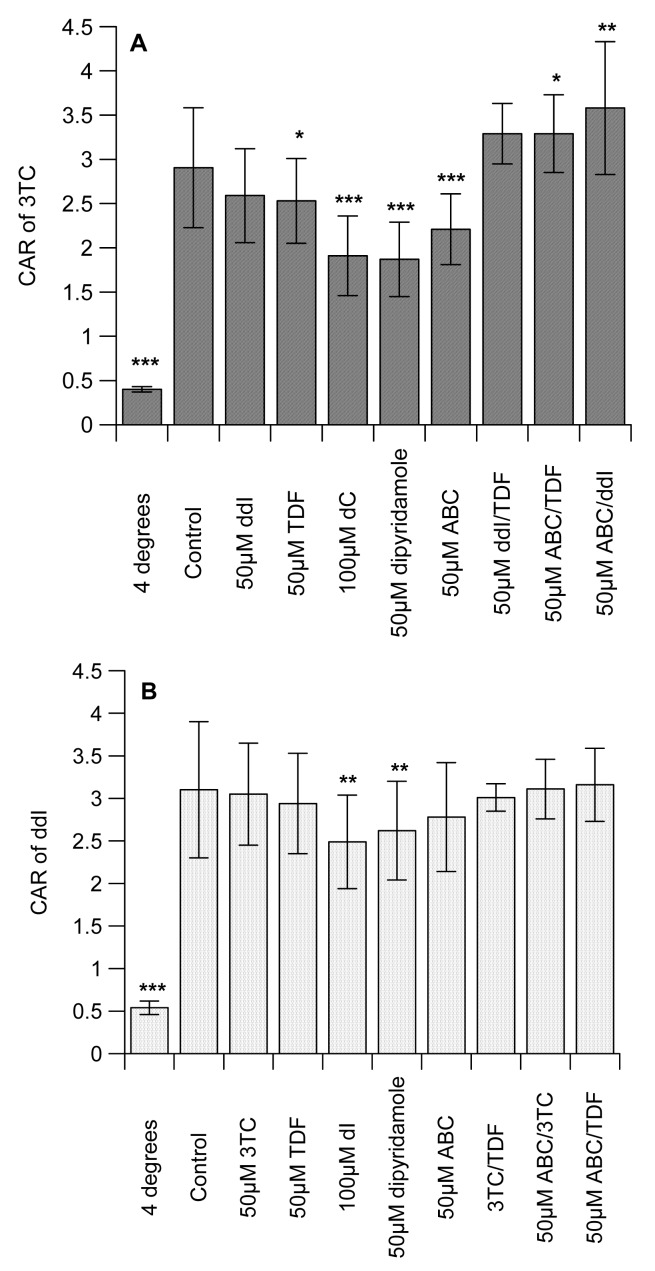

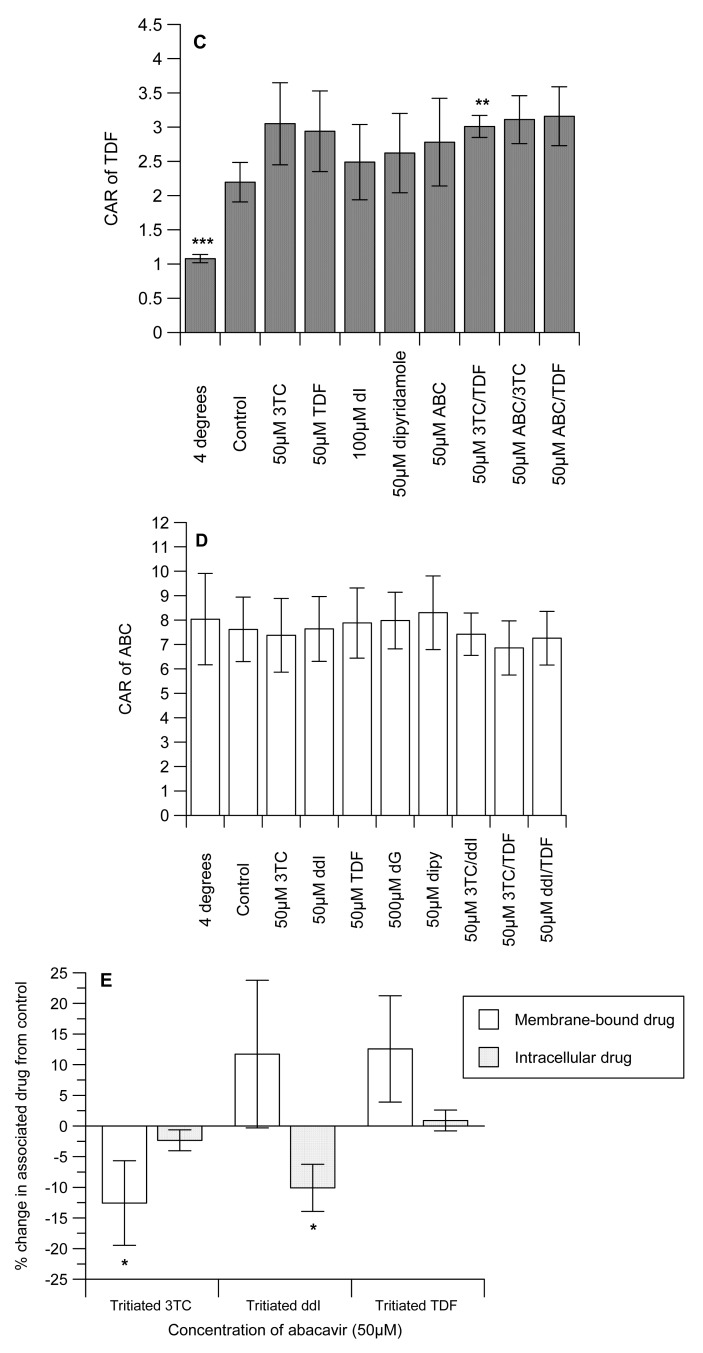
(**A**) The effects of drugs (alone or in combination) on the CAR of [^3^H]-3TC; **(B**) [^3^H]-ddI; (**C**) [^3^H]-TDF, (**D**) [^3^H]-ABC and (**E**) effects of 50 μM ABC on membrane-bound and intracellular [^3^H]-3TC, -ddI and -TDF in PBMCs isolated from blood buffy coats. Isolated PBMCs (5 × 10^6^ cells) were incubated (30–45 min, 4 °C, or 37C °C) in the absence or presence of the drugs (at concentrations indicated) before the assays were terminated as described in the methods section. Each bar represents mean ± SEM. *P < 0.05, **P < 0.01, ***P < 0.001 compared to control; n = 11. dC, deoxycytidine; dI, deoxyinosine; dG, deoxyguanosine.

Table 1 shows the octanol-saline partition coefficient for [^14^C]-mannitol (control), [^3^H]-3TC, -ddI, -TDF and -ABC, with the following rank order: with ABC ≫ 3TC ≥ ddI > TDF (Table 1). Preliminary studies examined the concentration-dependent effects of the unlabelled interacting drugs on the intracellular accumulation of the labelled drugs in CEM, its variant cells and U937 cell lines. Of all the compounds, only [^3^H]-ddI showed any differential accumulation in the CEM and its variant cells (data not shown). We observed no concentration-dependent effects of the unlabelled interacting drugs (3TC, ddI, TDF and ABC) on the accumulation of the labelled drugs in CEM, CEM_VBL_, CEM_E1000_ and U937 cells (data not shown). We observed that the cell-associated concentration of 3TC, ddI and TDF were temperature-sensitive, being significantly (*P* < 0.001) reduced at 4 °C (Figure 1A, 1B and 1C). In contrast, incubation at 4 °C did not affect the concentration of [^3^H]-ABC. The drugs displayed differential association, being highest in [^3^H]-3TC and least in [^3^H]-TDF (7.62 ± 1.32 ≫ 3.10 ± 0.8 > 2.91 ± 0.68 > 2.20 ± 0.29 for [^3^H]-ABC, -ddI, -3TC and -TDF, respectively).

The CAR of 3TC was unaffected by ddI, but TDF significantly (*P* < 0.05) reduced uptake (Figure 1A). Similarly, ABC significantly (*P* < 0.001) decreased the CAR of [^3^H]-3TC. As expected, the cytidine analogue, dC and the inhibitor of nucleoside transporter systems, dipyridamole significantly (*P* < 0.0001) reduced the CAR of [^3^H]-3TC. Manipulations that investigated the effects of ddI/TDF did not alter the CAR of [^3^H]-3TC, but both ABC/TDF and ABC/ddI significantly (*P* ≤ 0.05) increased the accumulation of [^3^H]-3TC. 3TC, TDF, ABC, 3TC/TDA, ABC/3TC and ABC/TDF had no effect on the accumulation of [^3^H]-ddI. However, dI (an inosine analogue) and dipyridamole significantly (*P* < 0.01) reduced the CAR of [^3^H]-ddI (Figure 1B). Of the drug incubations tested only 3TC/TDF significantly (*P* < 0.05) reduced the CAR of [^3^H]-TDF (Figure 1C). As TDF is not a nucleoside analogue, its accumulation was unaffected by dI or dipyridamole. None of the manipulations altered the accumulation of [^3^H]-ABC (Figure 1D).

Given the marked inhibitory effect of ABC on the accumulation of [^3^H]-3TC (Figure 1A), subsequent studies investigated its effects on membrane-bound and intracellular drug levels. ABC significantly (*P* < 0.05) decreased the membrane-bound and intracellular drug concentrations of both [^3^H]-3TC and -ddI, respectively. In contrast, intracellular [^3^H]-3TC, membrane-bound [^3^H]-ddI and both membrane-bound and intracellular [^3^H]-TDF were unaffected by ABC (Figure 1D).

The average levels of P-gp, MRP1 and BCRP were 0.61 ± 0.23, 16.94 ± 5.03 and 0.55 ± 0.14, respectively, but there was no relationship between individual transporter expression for each PBMC sample and the CAR values for any drug.

### 3.2. Discussion

The rapidity (within 12 weeks of treatment start) with which treatment failure develops and the emergence of resistance mutations is surprising for the highly potent three drug combinations used in TDF-containing regimens. In order to find a mechanistic basis for this, we studied an important aspect of intracellular drug accumulation (*i.e.*, lipophilicity), used primary human cells to investigate nucleoside/nucleotide interactions at the level of transport and also examined the effects of drug transporters on the accumulation of the compounds under investigation.

The octanol-saline partition coefficient determined for [^14^C]-mannitol was similar to reported values [24]. We observed differential lipophilicity of the drugs with ABC being the most lipophilic and tenofovir the least (Table 1). The accumulation of all of the compounds was inhibited by incubation at 4 °C, but thie manipulation did not affect the accumulation of [^3^H]-ABC (Figure 1D) due to its highly lipophilic nature. Interestingly, the rank order of accumulation of the drugs is identical to their lipophilicity.

Although there is some evidence that some NRTIs/NtRTI are substrates of P-gp, BCRP and MRP [8-19], we observed that of the compounds tested, only [^3^H]-ddI may be a P-gp substrate (data not shown). Our observation that [^3^H]-3TC is not a P-gp substrate is in agreement with published literature [23].

As evidence shows that some of the compounds under investigation are substrates of influx transporters such as hOATs and hOCTs [14,24-26], we postulate that they may compete for cellular entry which will consequently reduce the accumulation of the measured NRTI or NtRTI. Reciprocal to this, other studies have shown that ABC, 3TC and TDF inhibit the activities of P-gp and MRP [20-22] which would increase the accumulation of the labelled interacting drug. Here, we observed that the positive control inhibitors (dC and dipyridamole) significantly (*P* < 0.001) reduced the accumulation of [^3^H]-3TC). However, we observed no evidence in our preliminary studies that [^3^H]-3TC is transported by P-gp. Therefore, the observation that TDF and ABC separately (*P* ≤ 0.05) reduced the CAR of [^3^H]-3TC, suggests the potential of these drugs to inhibit the influx of [^3^H]-3TC or their direct competition with [^3^H]-3TC for influx (Fig 1A). We postulate that the extensive lipophilicity and accumulation of ABC may have reduced the capacity of the cells to accumulate [^3^H]-3TC. ABC/TDF and ABC/ddI significantly (*P* ≤ 0.05) increased the accumulation of [^3^H]-3TC, but not ddI/TDF. This observation is favourable in explaining the rationale for triple drug combination; its reveals that these drug-drug interactions at the transporter level cannot explain the treatment failures seen with TDF, ABC and 3TC. Evidence suggests that ABC inhibits BCRP, but TDF and ddI are devoid of any BCRP inhibitory effects [21]. Thus as ddI does not inhibit P-gp or MRP activity [23], we postulate that the possible inhibitory effects of the interacting drugs (ABC/TDF and ABC/ddI) on BCRP which mediates the efflux of 3TC [10, 19] may be responsible for the increase in accumulation of [^3^H]-3TC.

Previous observations (examined using guinea-pig brain perfusion and choroid plexus models) have shown increased intracellular accumulation of [^3^H]-ddI by ABC [25], which was shown to inhibit P-gp [20]. Although we observed that ddI may be a P-gp substrate, there was lack of interaction between TDF, ABC, 3TC and [^3^H]-ddI (Figure 1B). However, our observation complements previous studies, which showed no significant interaction between TDF and ddI in primary human cells [35]. As expected, the positive controls (dI and dipyridamole) significantly (*P* < 0.001) reduced the accumulation of [^3^H]-ddI.

None of the drug incubations decreased the accumulation of [^3^H]-TDF. The increase in the CAR of [^3^H]-TDF by 3TC/ABC cannot explain the emergence of treatment failure. However, this observation somewhat corroborates the observations of no adverse intracellular drug interaction between TDF and ABC that could explain the suboptimal viral response in patients treated with TDF+ABC+3TC regimens [36]. Furthermore, the accumulation of [^3^H]-ABC was not altered by any of the manipulations, suggesting that the extensive lipophilicity of the [^3^H]-ABC over rides the effect of any drug–drug interactions.

Given the marked reduction in 3TC accumulation obtained with ABC-treated samples we further studied its effects on membrane-bound and intracellular drug concentrations on [^3^H]-3TC, -ddI and -TDF. These studies revealed a modest, but significant, reduction in membrane-bound and intracellular drug concentrations of both [^3^H]-3TC and -ddI, respectively, suggesting the potential for ABC to reduce the cellular concentration of co-administered NRTIs and NtRTI.

Except for [^3^H]-ddI, we observed no evidence that the drugs are substrates of the drug efflux transporters (P-gp, MRP and BCRP); and there was no correlation between the accumulation of the drugs to any single transporter. The high lipophilicity of ABC may explain its observed effects. Overall, we observed some dug-drug interactions which may alter the concentrations of the active phosphorylated metabolites and potentially contribute to the emergence of resistance. Therefore, it may be important to focus on drug–drug interactions and the possible effects of drug-food interactions on changes in the intracellular phosphorylated metabolites of the drugs.

## 4. Conclusions

Although TDF and ABC separately appeared to limit cell-associated accumulation of 3TC, inconsistent findings were noted when both drugs were co-incubated. The impact of drug–drug interactions and the possible effects of drug-food interactions on levels of phosphorylated metabolites warrant further study.

## Figures and Tables

**Table 1.** Octanol-saline partition coefficients of the drugs.

**Drugs**	**Partition coefficient (mean ± SD)**

[^14^C]-Mannitol (control)	0.0022 ± 0.0001
[^3^H]-Lamivudine	0.117 ± 0.0049
[^3^H]-Didanosine	0.058 ± 0.0028
[^3^H]-Tenofovir	0.0061 ± 0.0004
[^3^H]-Abacavir	7.065 ± 0.44

## Abbreviations

NRTI: nucleoside reverse transcriptase inhibitor
NtRTI: nucleotide reverse transcriptase inhibitor
dC: deoxycytidine
dI: deoxyinosine
dG: deoxyguanosine
